# Convergence Gas Sensors with One-Dimensional Nanotubes and Pt Nanoparticles Based on Ultraviolet Photonic Energy for Room-Temperature NO_2_ Gas Sensing

**DOI:** 10.3390/nano13202780

**Published:** 2023-10-17

**Authors:** Sohyeon Kim, Ju-Eun Yang, Yoon-Seo Park, Minwoo Park, Sang-Jo Kim, Kyoung-Kook Kim

**Affiliations:** 1 Department of IT Semiconductor Convergence Engineering, Research Institute of Advanced Convergence Technology, Tech University of Korea, Siheung 15073, Republic of Korea; 2 School of Material Science and Engineering, Gwangju Institute of Science and Technology, Gwangju 61005, Republic of Korea

**Keywords:** NO_2_ gas sensor, ZnO, nanorod, nanotube, metal nanoparticles

## Abstract

Zinc oxide (ZnO) is a promising material for nitrogen dioxide (NO_2_) gas sensors because of its nontoxicity, low cost, and small size. We fabricated one-dimensional (1D) and zero-dimensional (0D) convergence gas sensors activated via ultraviolet (UV) photonic energy to sense NO_2_ gas at room temperature. One-dimensional ZnO nanorod (ZNR)-based and ZnO nanotube (ZNT)-based gas sensors were synthesized using a simple hydrothermal method. All the sensors were tested under UV irradiation (365 nm) so that they could be operated at room temperature rather than a high temperature. In addition, we decorated 0D Pt nanoparticles (NPs) on the gas sensors to further improve their sensing responsivity. The NO_2_-sensing response of the ZNT/Pt NP convergence gas sensor was 2.93 times higher than that of the ZNR gas sensor. We demonstrated the complex effects of UV radiation on 1D ZnO nanostructures and 0D metal nanostructures in NO_2_ gas sensing.

## 1. Introduction

In recent years, the monitoring of air pollutant emissions has become significantly important because of severe air pollution and global warming. Nitrogen oxide (NO_x_) gases, such as nitric oxide (NO) and nitrogen dioxide (NO_2_), are air pollutants derived from the combustion of nitrogen and oxygen in fossil fuels and motor vehicles. NO_2_ reacts with the atmosphere to form ozone and contributes to the formation of photochemical smog and acid rain [1,2]. Furthermore, even at low concentrations, prolonged exposure to NO_2_ gas can cause fatal diseases [3]. Therefore, the development of NO_2_ gas sensors with high responsivity and stable operational characteristics is essential.

Gas-sensing mechanisms are derived from electron transfer between target gas molecules and the oxygen species adsorbed on the surface of sensing materials, and this process is related to the surface area, structure, and electron properties of sensing materials [4]. Thus, it is important to increase the surface area of sensors because their sensing responsivity depends on surface reactions. In the case of one-dimensional (1D) nanostructured materials with a large surface-to-volume ratio, the surface phenomenon is more dominant than that in thin-film-structured sensors. Therefore, 1D-nanostructured gas sensors have attracted considerable attention owing to their high sensing responsivity [5,6,7]. Among 1D structures, nanorods (NRs) with large surface area have been widely studied as gas sensors. Although nanotubes (NTs) appear to be more suitable as sensors than NRs because of their larger surface area, there are few studies on NT gas sensors. Therefore, in this study, we selected vertically aligned 1D NTs as gas sensors to improve sensing responsivity via increasing surface area. Moreover, according to our previous work [8,9], sensing responsivity increases when metal nanoparticles (NPs) are added to NTs owing to spillover and localized surface plasmon resonance (LSPR) effects.

Metal–oxide semiconductor (MOS) materials are preferred for gas-sensing devices owing to the ease with which they can be processed and their high gas response and high stability [10,11]. Among MOS materials, zinc oxide (ZnO) is considered a promising material for gas sensors because of its non-toxicity, low cost, and small dimensions. ZnO can be synthesized though various methods, such as sputtering [12], metalorganic vapor-phase epitaxy [13], thermal chemical vapor deposition [14,15], and hydrothermal methods [7,16]. In particular, the hydrothermal methods have a lot of advantages, including cost-effectiveness, low process temperatures, and large-scale production. Furthermore, another reason why ZnO is attracting attention is that ZnO nanostructures can be easily fashioned into nanowires (NWs), NRs, NTs, and nanobelts [17,18,19]. ZnO-based NO_2_ gas sensors have been extensively studied owing to these advantages [20,21,22,23,24,25,26]. Conventional ZnO-based gas sensors typically operate at high temperatures of 300–500 °C because sufficient thermal energy is required for surface reactions [27]. However, this negatively affects their stability and durability owing to the diffusion and sintering effect at the ZnO grain boundaries [17,28]. Therefore, a ZnO-based gas sensor with high responsivity at low operating temperatures should be developed.

Recently, research on gas sensors capable of detecting target gases at room temperature (RT) has extended. To operate gas sensors at RT, several studies on ZnO-based gas sensors using ultraviolet (UV) light have been reported. Meng et al. [29] reported a single-sheet ZnO nanostructure-based gas sensor operating at RT using a 365 nm UV lamp. Kwon et al. [7] reported a RT-operable ZnO/TiO_2_ NR-based gas sensor that was developed using a 370 nm UV light-emitting diode (LED). Cai et al. [8] reported a ZnO NW-based gas sensor under 365 nm UV light that was capable of detecting target gases at RT. The above studies indicate that UV activation is a promising strategy for operating ZnO-based gas sensors at RT. When ZnO-based gas sensors are exposed to light with an energy equal to or higher than the bandgap of ZnO, the electrons in the valence band are excited and move to the conduction band, and electron–hole pairs are generated in the ZnO surface region. The remaining photogenerated electrons reduce the depletion layer region and increase the current of ZnO-based gas sensors, thereby allowing for gas sensing at RT.

In this study, we prepared 1D and zero-dimensional (0D) convergence gas sensors activated via UV photonic energy capable of detecting NO_2_ at room temperature. One-dimensional ZnO NR (ZNR) and ZnO NT (ZNT) gas sensors with large surface-to-volume ratios were synthesized using a simple hydrothermal method and selective etching. In most previous ZNT gas sensor studies, ZNT synthesis was carried out using electrospinning and thermal chemical vapor deposition methods, and the ZNTs synthesized using these methods had a horizontally arranged structure. [14,17,30,31]. The 1D ZNRs and ZNTs synthesized via one-step hydrothermal methods in this study are vertically aligned, allowing their surface area to be maximized through the control of their diameters and lengths. In addition, we decorated randomly sized 0D Pt NPs on the 1D ZNR-based and ZNT-based gas sensors, which are 1D and 0D convergence gas sensors, and proved that Pt NPs improved the NO_2_ gas-sensing performance of the ZnO-based gas sensors. To increase the NO_2_ gas-sensing efficiency of the sensors at RT, we used a UV emitter (365 nm) for photoactivation rather than a high-temperature operation. We demonstrated that the wall structures of the ZNTs constituted the major reason for the higher responsivity of the ZNT gas sensors compared to the ZNR gas sensors. In addition, UV light had complex effects on 1D ZnO nanostructures and 0D metal nanostructures in NO_2_ gas sensing.

## 2. Materials and Methods

### 2.1. ZNR and ZNT Synthesis

**Figure 1:**
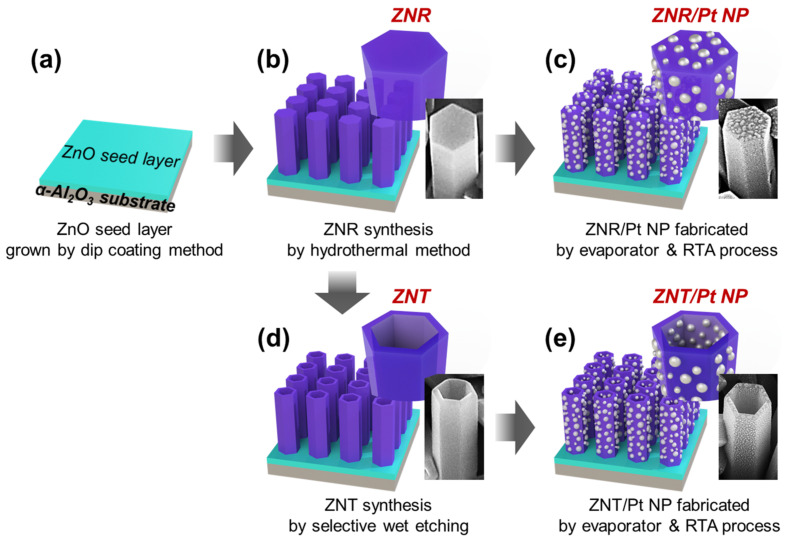
Fabrication procedure for ZNR-based and ZNT-based gas sensors. Schematics of (**a**) ZnO seed layer grown on sapphire substrate, (**b**) ZNR-based gas sensor, (**c**) ZNR/Pt NP-based gas sensor, (**d**) ZNT-based gas sensor, and (**e**) ZNT/Pt NP-based convergence gas sensor.

Figure 1 shows the schematics of the ZnO seed layer, the ZNRs, ZNTs, ZNRs covered with Pt NPs (ZNRs/Pt NPs), and the ZNTs covered with Pt NPs (ZNTs/Pt NPs). To form the ZnO seed layer (Figure 1a), an aqueous solution of 0.25 mM of zinc acetate (Zn(C_2_H_3_O_2_)_2_) in deionized (DI) water was prepared via stirring for 20 min. Then, a piece of a sapphire substrate was immersed in the solution. A ZnO seed layer was synthesized on the sapphire substrate at 90 °C for 1 h. Subsequently, ZNRs were grown on the ZnO seed layer in a 300 mL aqueous solution of 6.7 mM of zinc nitrate hexahydrate (Zn(NO_3_)_2_∙6H_2_O) and 2.2 mM of hexamethylenetetramine (C_6_H_12_N_4_) in DI water at 90 °C for 3 h, as shown in Figure 1b. ZNT arrays were formed through the selective wet etching of ZNRs via decreasing the solution temperature, as shown in Figure 1d. The temperature was decreased from 90 °C to 40 °C and then maintained at 40 °C in the solution for 3 h for the selective etching process (Figure S1). The ZNR and ZNT samples were annealed at 600 °C in ambient air for 1 h.

### 2.2. Pt NP Formation

A Pt layer was deposited on the ZNRs and ZNTs using a DC sputtering system before the annealing process at a base pressure of 0.09 mbar, current of 10 mA, and deposition time of 200 s. The samples were annealed at 600 °C in ambient air for 15 min. The Pt layer was transformed into Pt NPs via agglomeration on the surfaces of the ZNRs and ZNTs, as shown in Figure 1c and e, respectively.

### 2.3. Gas Sensor Material Characterization

The morphological and structural properties of the samples were observed using a field-emission scanning electron microscope (FE-SEM, Hitachi S4300, Hitachi Ltd., Chiyoda City, Tokyo) with an acceleration voltage of 10 kV and a working distance of 15 mm and a field-emission transmission electron microscope (FE-TEM, JEM-2100F, JEOL Ltd., Akishima, Tokyo) operated at 300 kV. Selected-area electron diffraction (SAED) analysis was also performed.

### 2.4. Gas Sensor Characterization

To fabricate resistive NO_2_ sensors, a pair of titanium (Ti, 100 nm) and gold (Au, 50 nm) electrodes with a gap of 5 mm was deposited on the ZNR, ZNT, ZNR/Pt NP, and ZNT/Pt NP gas sensors (total device size: 15 × 10 mm^2^; size of one electrode pad: 5 × 10 mm^2^).

The NO_2_ gas-sensing responses were measured using a homemade gas chamber connected to two mass flow controllers for NO_2_ gas and air, which controlled the NO_2_ gas concentration (Figure S2). NO_2_ gas was injected into the chamber after the atmosphere in the chamber was stabilized by injecting forming air into the chamber for 30 min. The change in the sensor resistance upon exposure to the target gas was measured using a source meter (Keithley 2400, Keithley Instruments Inc., Cleveland, OH, USA) with a custom-written LabView program. All measurements were conducted under 365 nm UV radiation at RT. In addition, the wavelength of the UV emitter suitable for the activation of the gas sensor was examined by conducting a finite-difference time-domain (FDTD) simulation using Lumerical. The sensor response, R, was defined as the ratio of the resistance in NO_2_ gas (R_g_) to that in air (R_a_): (R_g_ − R_a_)/R_a_.

## 3. Results and Discussion

### 3.1. Characterization of ZNRs and ZNTs

**Figure 2:**
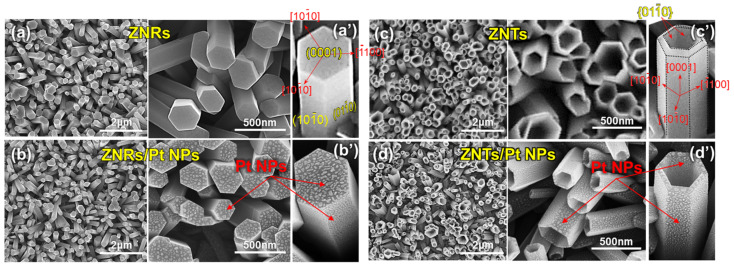
FE-SEM images of (**a**) ZNRs, (**b**) ZNRs covered with Pt NPs (ZNRs/Pt NPs), (**c**) ZNTs, and (**d**) ZNTs covered with Pt NPs (ZNTs/Pt NPs). (**a′**–**d′**) High-magnification FE-SEM images of ZNRs, ZNRs/Pt NPs, ZNTs, and ZNTs/Pt NPs, respectively.

Figure 2a shows the FE-SEM images of the hexagonal ZNR arrays synthesized using the hydrothermal method. The diameters of the ZNRs were within the range of 150–550 nm, and the height of the ZNRs was approximately 1.4 μm. As shown in Figure 2a′, the ZnO (0001) plane was formed on the tops of the ZNRs, and the ZnO (10-10) and (01-10) planes were formed on their sidewalls. After Pt layer deposition on the ZNRs and annealing at 600 °C (Figure 2b), Pt NPs formed on the surface because the as-deposited Pt nano-layer is generally metastable and transforms into an energetically stable droplet form even at a temperature below its melting point [32,33]. As shown in Figure 2b′, the top and sidewalls of the ZNRs were covered with Pt NPs. Figure 2c shows the ZNTs obtained after the selective wet etching of the ZNRs. Hexagonal holes formed in the upper parts of the ZNTs, resulting in the formation of ZNT walls with a thickness of approximately 40 nm. The inner planes of the walls belonged to the {01-10} family of hexagonal planes (Figure 2c′). Figure 2d shows the ZNTs covered with Pt NPs, which were formed using the same process as the ZNRs/Pt NPs (Figure 2b). Pt NPs formed on the top, outer, and inner planes of the ZNTs (Figure 2d′). In addition, the energy-dispersive X-ray spectroscopy results confirmed that the ZNR/Pt NP and ZNT/Pt NP samples were composed of ZnO and Pt (Figure S3).

**Figure 3:**
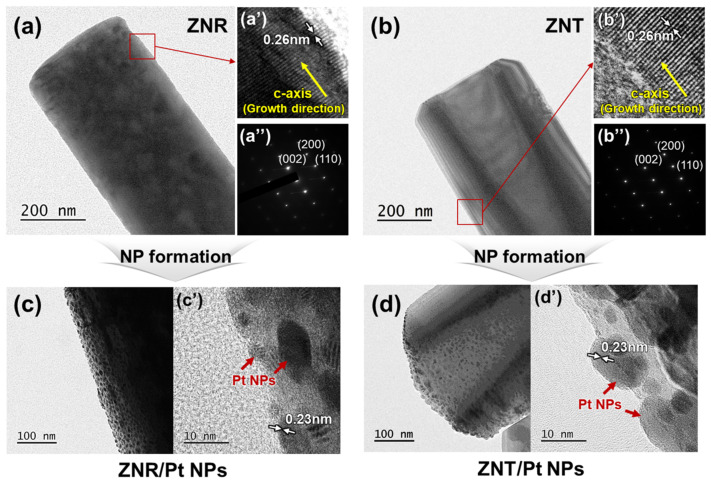
FE-TEM images of (**a**) ZNR, (**b**) ZNT, (**c**) ZNRs/Pt NPs, and (**d**) ZNTs/Pt NPs. High-magnification FE-TEM images show the average lattice spacings of (**a′**) ZNR, (**b′**) ZNT, and (**c′**,**d′**) Pt NPs formed on ZNRs and ZNTs. SAED patterns of (**a″**) ZNR and (**b″**) ZNT.

Further structural and material analysis was performed using the FE-TEM images and SAED patterns of individual ZNR and ZNT samples, as shown in Figure 3a,b, respectively. A selectively etched hole was made at the top of the ZNT. The high-magnification images (Figure 3a′,b′) confirm that the average lattice spacings of the ZNR and ZNT were 0.26 nm each, corresponding to the d space of the wurtzite ZnO (0002) plane. This indicates that the ZNRs and ZNTs had perfect crystal structures and grew along the *c*-axis. Figure 3a″,b″ show that the SAED patterns of the individual ZNR and ZNT samples exhibited distinct diffraction spots, indicating that the ZNR and ZNT had single-crystal structures. Figure 3c,d show the ZNR/Pt NP and ZNT/Pt NP samples after Pt NP formation on the ZNRs and ZNTs. Pt NPs were nonuniformly distributed on the ZNR and ZNT surfaces. Figure 3c′,d′ show that the average lattice spacings of the Pt NPs on the ZNRs and ZNTs were both 0.23 nm, which is in good agreement with the lattice spacing of the Pt (111) crystal plane.

### 3.2. FDTD Simulation

**Figure 4:**
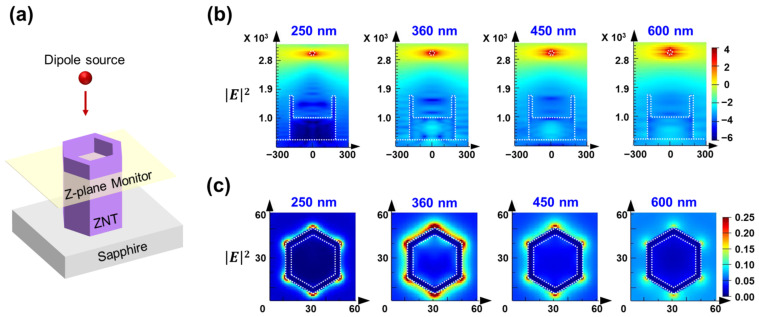
(**a**) Schematic of the FDTD simulation model. (**b**) Electric field intensities in Y-plane monitor. (**c**) Electric field intensities in Z-plane monitor.

A single-ZNT model was set up on a sapphire substrate, as shown in Figure 4a. The length, diameter, and wall thickness of the ZNT model were similar to the experimentally obtained values. Dipole sources with center wavelengths of 250, 360, 450, and 650 nm were placed above the ZNT. Monitors were set up to measure the electric fields in the Y-plane and Z-plane. A Z-plane monitor was placed within the ZNT to investigate the electric-field changes around the ZNT nanostructure. Figure 4b shows the distributions of the electric-field intensities at the cross sections of the ZNT. The strongest electric field and waveguide effects appeared on the surface and inside the ZNT structure when the dipole source with a center wavelength of 360 nm was used. The electric field close to the ZNT was negligible when the dipole source with a center wavelength of 250 nm was used. The electric fields close to the ZNT obtained using the 450 nm and 600 nm dipole sources were weaker than the field obtained using the 360 nm dipole source. Figure 4c shows the electric field distribution on the Z-plane monitor within the ZNT. At all wavelengths, the electric field around the ZNT became stronger when the light reached the ZNT. Similar to the trend in Figure 4b, the strongest electric field on the ZNT surface was observed in the case of the 360 nm dipole source. The strongest interaction between the sensor materials and structures was obtained at a wavelength of 360 nm in the FDTD simulation. This confirmed that light with a wavelength of approximately 360 nm maximized the photoactivation of the sensor material for gas sensing. Therefore, all gas-sensing measurements under UV irradiation were performed using a UV emitter with a wavelength of 365 nm, which is close to 360 nm.

### 3.3. NO_2_ Gas-Sensing Properties

**Figure 5:**
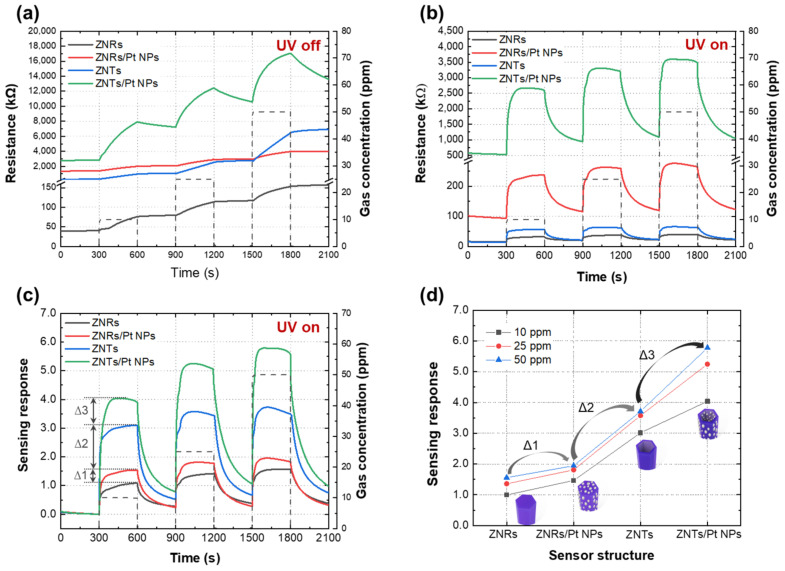
(**a**) Resistance of gas sensors at different NO_2_ gas concentrations in the dark and (**b**) under UV irradiation. (**c**) Gas-sensing response of sensors under UV irradiation. (**d**) Gas-sensing response according to the sensor nanostructures.

Figure 5a shows the NO_2_ gas-sensing results of the ZNR, ZNR/Pt NP, ZNT, and ZNT/Pt NP sensors at different gas concentrations (10 ppm, 25 ppm, and 50 ppm) in the dark. Variations in the gas-sensing response were observed for all the sensors. In particular, the resistances of the ZNT and ZNT/Pt NP sensors were significantly higher than those of the ZNR and ZNR/Pt NP sensors. However, no recovery properties were observed for the ZNR, ZNR/Pt NP, or ZNT sensors. Only the ZNT/Pt NP convergence gas sensors exhibited response and recovery without UV irradiation. Figure 5b shows the NO_2_ gas-sensing results obtained under UV irradiation. The basic resistances in air were lower than those measured in the dark owing to the photogenerated electrons on the ZnO surface under UV light. Gas-sensing response and recovery were observed under UV irradiation, and the gas-sensing response gradually increased with the NO_2_ concentration for all the sensors. Figure 5c shows the sensing response values calculated from the resistance results. The sensing response values at a NO_2_ concentration of 25 ppm for the ZNR, ZNR/Pt NP, ZNT, and ZNT/Pt NP sensors were 1.4, 1.8, 3.6, and 5.5, respectively. The ZNT/Pt NP sensor had the highest response value of 5.8 at a NO_2_ concentration of 50 ppm. The responsivity of the ZNT-based gas sensors was higher than that of the ZNR-based gas sensors. Furthermore, the sensing responses of the Pt-NP-covered ZNR and ZNT sensors were better than those of the ZNR and ZNT sensors, respectively. These results are similar to those reported by Young et al. [34], where the addition of Pt NPs to ZNRs improved the methanol-gas-sensing responsivity. Figure 5d shows the sensing responses based on the gas sensor structures. The changes in the sensing response as the structure changed from ZNRs to ZNRs/Pt NPs, ZNRs/Pt NPs to ZNTs, ZNRs to ZNTs, ZNTs to ZNTs/Pt NPs, and ZNRs/Pt NPs to ZNTs/Pt NPs are denoted by ∆1, ∆2, (∆1 + ∆2), ∆3, and (∆2 + ∆3), respectively. It should be noted that (∆2 + ∆3) and (∆1 + ∆2) were the highest and second highest, respectively, which implies that the changes in the shape of the nanostructure had the strongest effect on the sensing response (Figure S4). Furthermore, ∆3 was higher than ∆1, which indicates that the effect of the Pt NPs on the ZNTs was stronger than that on the ZNRs.

### 3.4. Effects of UV Irradiation and Pt NP Addition on Gas Sensing

**Figure 6:**
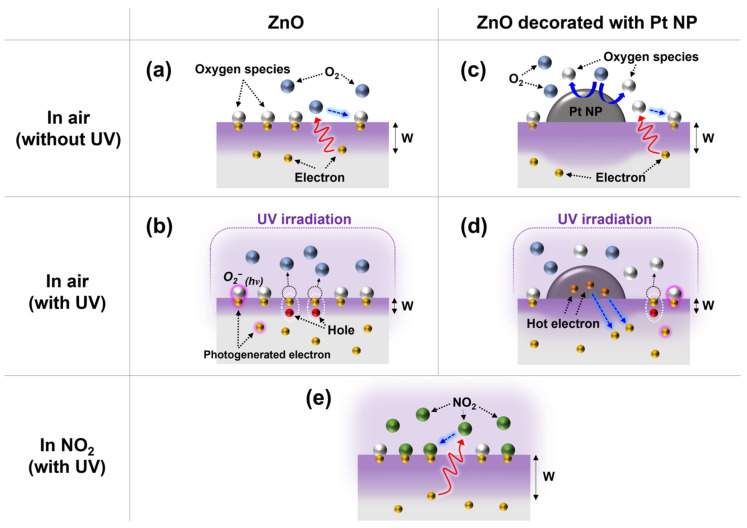
RT-operated NO_2_-gas-sensing mechanism under UV irradiation. (**a**) Oxygen species are chemisorbed on the surface in air. (**b**) Electron–hole pairs are photogenerated under UV light. (**c**) In the case of Pt NP modification, the Pt NPs on the ZnO surface enhance surface reactions in air due to the spillover effect. (**d**) Hot electrons are generated in Pt NPs and injected into ZnO. (**e**) NO_2_ gas reacts with oxygen species chemisorbed on the ZnO surface.

Figure 6 shows the NO_2_-sensing mechanism of the ZnO gas sensors under UV irradiation. Figure 6a,b show the surface reactions of ZnO without Pt NPs. As shown in Figure 6a, when the ZnO gas sensors were exposed to atmospheric air, the electrons in the conduction band of ZnO were transferred to the adsorbed oxygen atoms. Subsequently, oxygen species were chemisorbed onto the ZnO surface.

O_2(g)_ ↔ O_2(ads)_ (1)

O_2(ads)_ + e^−^ → O^2−^_(ads)_ (2)

Consequently, the width of the surface depletion layer and the resistance of the ZnO gas sensor increased because of the consumption of electrons in the ZnO surface region. Under UV irradiation (Figure 6b), electron–hole pairs were generated in the ZnO surface region. The photogenerated holes reacted with the negatively charged chemisorbed oxygen species, generating oxygen atoms.

*hv* → *h*^+^_(*hv*)_ + e^−^_(*hv*)_ (3)

*h*^+^_(*hv*)_ + O^2−^_(ads)_ → O_2(g)_ (4)

Meanwhile, photogenerated electrons combined with ambient oxygen, creating additional photoinduced oxygen species, which were bound by weaker forces compared to the chemisorbed oxygen species.

e^−^_(*hv*)_ + O_2(g)_ → O^2−^_(*hv*)_ (5)

Although some of the photogenerated electrons were consumed during the production of oxygen species, the width of the surface depletion layer and the resistance of ZnO decreased because of the large number of photogenerated carriers on the surface. As shown in Figure 6e, when the ZnO gas sensor was exposed to NO_2_ gas, as shown in Figure 6e, the NO_2_ gas reacted with both photoinduced oxygen species and photogenerated electrons at RT, thereby consuming electrons in the ZnO surface region. Subsequently, NO_2_ gas was adsorbed onto the ZnO surface.

NO_2(g)_ + e^−^ → NO^2−^_(ads)_ (6)

NO_2(g)_ + O^2−^_(*hv*)_ + 2e^−^ → NO^2−^_(ads)_ + 2O^−^_(ads)_ (7)

Consequently, the width of the surface depletion layer and the resistance of ZnO increased. The Pt NPs enhanced the sensing responses of the ZNR/Pt NP and ZNT/Pt NP convergence gas sensors owing to the spillover and LSPR effects. Figure 6c shows the spillover effect of Pt NPs on the ZnO surface. Here, the Pt NPs acted as catalysts, which enhanced the dissociation of oxygen molecules to oxygen species such as O^−^, O^2−^, and O_2_^−^. In this process, electrons were captured, and the width of the depletion layer increased at the interface between ZnO and the Pt NPs. Consequently, the surface reactions were enhanced because O^−^ is more chemically active than O_2_^−^.

As shown in Figure 6d, under UV irradiation, the hot electrons generated in the Pt NPs were injected into the conduction band of the ZnO. The hot electrons could not be transferred back to the Pt NPs from the ZnO because of the Schottky barrier formed at the interface between the Pt NPs and ZnO (Figure S5). The migration of photogenerated electrons on the ZnO surface enhanced surface reactions and gas-sensing responses. Consequently, the addition of Pt NPs to the ZNRs and ZNTs improved the gas-sensing responses of the sensors.

### 3.5. Effect of Tube Structures on Gas Sensing

**Figure 7:**
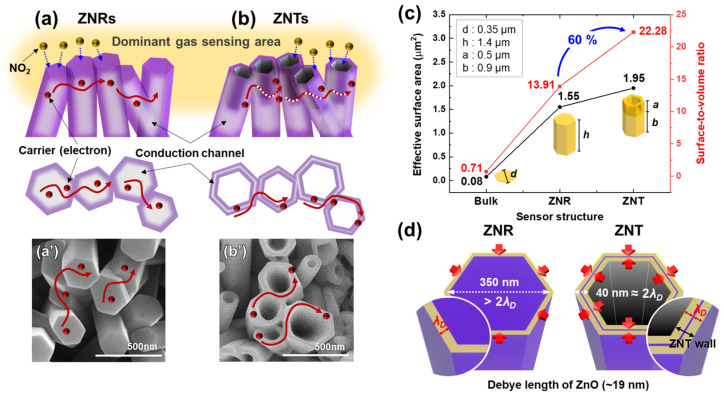
(**a**,**a′**,**b**,**b′**) Schematic and FE-SEM images of ZNRs and ZNTs that are vertically aligned in random directions. (**c**) Effective surface area for gas sensing and surface-to-volume ratio depending on the sensor structures. (**d**) Schematics for comparing sizes and Debye length of fabricated ZnO nanostructures.

The structural change in ZnO was the greatest factor for improving sensing responsivity, although the Pt NPs partially contributed to the improvement in responsivity (Figure 5). One of the reasons for this improvement in responsivity due to the structural change from ZNRs to ZNTs was the increase in the effective surface area of the sensors, on which NO_2_ gas can react with chemisorbed oxygen species. Figure 7a shows schematics and SEM images of the ZNRs and ZNTs grown using the wet process for comparison. The ZNRs and ZNTs were arranged vertically and randomly, as shown in the SEM images in Figure 7a′,b′, respectively. NO_2_ gas was injected from the top of the vertical ZnO nanostructures (Figure 7a,b). This procedure indicated that the upper regions of the ZNRs and ZNTs were dominant in gas sensing and that gas-sensing reactions occurred in additional regions on the inner walls of the ZNTs. Figure 7c shows the increase in the effective surface area caused by the structural changes. In bulk or thin-film gas sensors, only the top surface provides an effective surface area for sensing. However, the effective surface area for the sensing activity considerably increases in 1D nanostructures. The surface area of the ZNR with a diameter of 350 nm and a length of 1.4 nm was 1.55 μm^2^, and that of the ZNT with the same diameter and length and a 500 nm deep hexagonal hole was 1.95 μm^2^. The surface-to-volume ratios of the bulk ZnO, ZNRs, and ZNTs were 0.71, 13.91, and 22.28, respectively. The surface-to-volume ratio of the ZNTs was 60% higher than that of the ZNRs. A large surface-to-volume ratio and high effective surface area improved the gas-sensing responsivity of the ZNTs owing to sufficient surface reactions.

Another reason for the enhanced gas-sensing responsivity was related to the depletion layer and Debye length (*λ_D_*). The gas-sensing response depends on the change in the surface resistance caused by the adsorption and desorption of NO_2_ gas. Therefore, the width of the surface depletion layer, which strongly affects surface resistance, can represent the effective gas-sensing range of MOS gas sensors. The width of the surface depletion layer typically depends on the Debye length

(8) $$\lambda_{D}=\sqrt{\frac{\varepsilon kT}{q^{2}n_{c}}}$$

where *ε* is the dielectric constant, *q* is the electric charge, and *n_c_* is the carrier density. According to a previous study, the gas-sensing responsivity of a nanostructure significantly improves when its size is close to or equal to the Debye length [35]. A related study has shown that the Debye length of ZnO is approximately 19 nm at RT [36]. One-dimensional nanostructures with cylindrical bodies can be fully depleted and controlled by their surface state when their diameter is close to or equal to twice their Debye length. In other words, when the diameter of the ZNRs was approximately 38 nm (twice the Debye length of ZnO), their gas-sensing responsivity was significantly enhanced because the resistance of the ZNRs was controlled by surface reactions. However, as shown in Figure 7d, the diameter of the ZNRs fabricated in this study was more than twice the Debye length. In the case of the ZNTs, the thickness of the ZNT walls was approximately 40 nm, which was close to 38 nm. Consequently, the ZNT-based gas sensors were more sensitive than the ZNR-based gas sensors.

In addition, as shown in Figure 5d, the Pt NPs significantly improved the responsivity of the ZNTs compared to the ZNRs. This was also due to the tube structure of the ZNTs, which consisted of walls with dimensions smaller than the diameters of the ZNRs. When Pt NPs formed on the ZnO surface, electrons moved from Pt to ZnO, and the width of the ZnO surface depletion layer increased regardless of the Debye length (Figure 6c). Therefore, the sensing responsivity of the ZNT/Pt NP sensors with thin ZNT walls (40 nm) was significantly affected by the increase in the depletion layer width and spillover effect (Figure S6). On the other hand, the sensing responsivity of the ZNR/Pt NP sensors was enhanced by the spillover effect, similar to the case of ZNT/Pt NP sensors. However, it was negligibly affected by the increase in the depletion layer width owing to the large diameter of the ZNRs (350 nm).

## 4. Conclusions

We prepared convergence gas sensors with 1D and 0D nanostructures activated via UV photonic energy capable of detecting NO_2_ gas with high responsivity at RT. The 1D ZNRs and ZNTs were fabricated using an all-solution process. To reduce the degradation of the gas sensors’ durability due to heat, all sensing measurements were conducted at RT with a UV emitter (365 nm) for the photoactivation of ZnO rather than using a high-temperature process. The sensing responses of the ZNR, ZNR/Pt NP, ZNT, and ZNT/Pt NP sensors at a NO_2_ concentration of 25 ppm were 1.4, 1.8, 3.6, and 5.5, respectively. The NO_2_-sensing performance of the ZNT/Pt NP convergence gas sensor was enhanced by the addition of Pt NPs and the ZnO tube structure. The Pt NPs that covered the ZNRs and ZNTs promoted the spilling of oxygen molecules and surface reactions as catalysts. In addition, the Pt NPs provided additional carriers for surface reactions via the LSPR effect induced using a UV emitter. Consequently, the gas sensors with Pt NPs exhibited better sensing responses than those without Pt NPs. Furthermore, the effect of the tube structure on the sensing responsivity was stronger than that of the Pt NPs. The NO_2_-sensing performance of the ZNT gas sensors with 40 nm thick hexagonal walls was better than that of the ZNR gas sensors owing to the smaller dimensions and larger effective surface area of the ZNTs compared to the ZNRs. The average surface-to-volume ratio of the ZNTs was 60% higher than that of the ZNRs. Therefore, the area available for NO_2_ gas detection increased, and the gas-sensing response improved. Moreover, the dimensions of the ZNT walls were similar to the Debye length of ZnO, which maximized the gas-sensing responsivity of the ZNT gas sensor at RT. In addition, we measured the gas-sensing responses of all the sensors to three additional gases (toluene, acetone, and methanol) (Figure S7). All the sensors showed high sensitivity only for NO_2_ gas and weak responsivity for the other three gases, thereby confirming the possibility of selective gas sensing.

## Data Availability

The data presented in this study are available on request from the corresponding author.

## Supplementary Materials

The following supporting information can be downloaded at: https://www.mdpi.com/article/10.3390/nano13202780/s1. Figure S1. Mechanism of selective etching of ZNR to form ZNT structure. Figure S2. The gas-sensing measurement system is illustrated in a schematic image. Figure S3. SEM-EDS results of ZNR/Pt NPs and ZNT/Pt NPs. Figure S4. Plot of change in gas-sensing responsivity according to structural changes. Figure S5. Energy bandgap diagram of ZnO and Pt in Schottky junction. Figure S6. Schematic images of increased depletion layer width of ZNRs and ZNTs due to Pt NPs formation. Figure S7. Sensing responsivity graph for NO_2_, acetone, toluene, and methanol gases in different gas concentrations. Ref. [37] is cited in Supplementary Materials.
