# Supplementary material for: Convergence Gas Sensors with One-Dimensional Nanotubes and Pt Nanoparticles Based on Ultraviolet Photonic Energy for Room-Temperature NO2 Gas Sensing
Source: Nanomaterials (Basel). 2023 Oct 17;13(20):2780. doi: 10.3390/nano13202780 (PMC10609275; doi:10.3390/nano13202780)
Supplement: Supplementary file 1 [file nanomaterials-13-02780-s001.zip › nanomaterials-2660576-supplementary.pdf]

Supplementary Materials

Convergence Gas Sensors with One-Dimensional Nanotubes and Pt Nanoparticles Based on Ultraviolet Photonic Energy for Room-Temperature NO_2 Gas Sensing

Sohyeon Kim [1], Ju-Eun Yang [1], Yoon-Seo Park [1], Minwoo Park [1], Sang-Jo Kim [2] and Kyoung-Kook Kim [1,*]

[1] Department of IT Semiconductor Convergence Engineering, Research Institute of Advanced Convergence Technology, Tech University of Korea, Siheung 15073, Republic of Korea

[2] School of Material Science and Engineering, Gwangju Institute of Science and Technology, Gwangju 61005, Republic of Korea

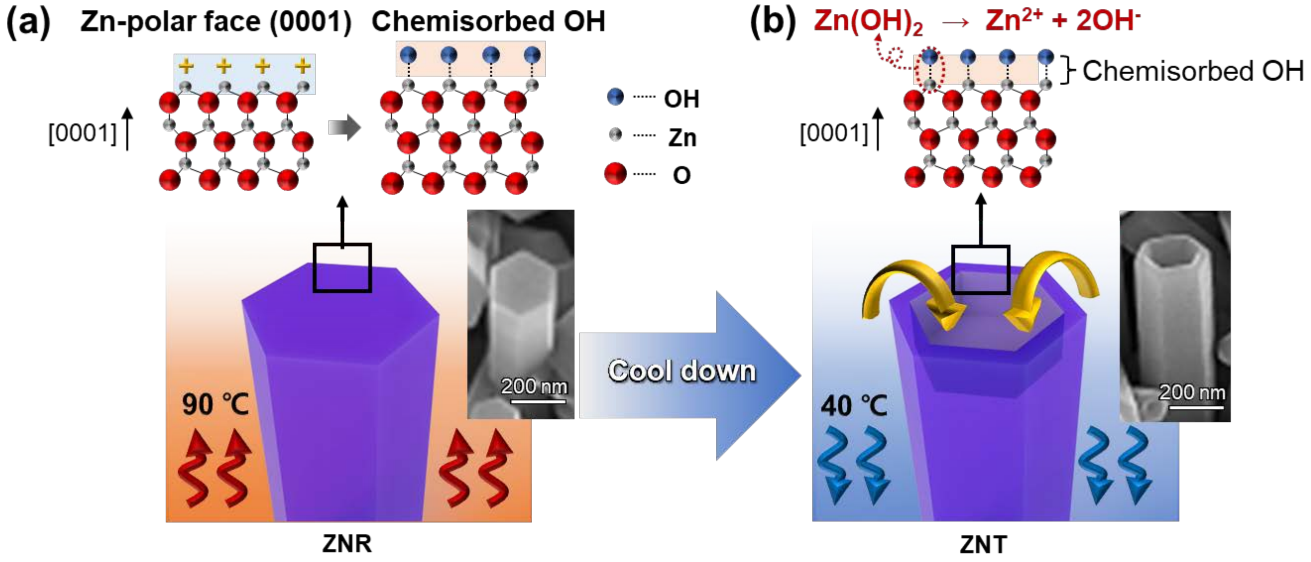

Figure S1. Mechanism of selective etching of ZNR to form ZNT structure. Schematic images of (a) ZNR synthesized by wet process and (b) tube formation with selective wet etching in the center of the ZNR. The Zn polar (0001) plane is exposed on top of the ZNR grown in [0001] direction, where OH^- ions are chemisorbed by dangling bonds of Zn ions, and a chemisorbed OH layer is formed on the top surface of the ZNR. ZnO was synthesized using this chemisorbed OH layer at a process temperature of 90 °C, as shown in Figure S1a. However, Zn polar face of ZnO is selectively etched by chemisorbed OH layer at a process temperature of 40 °C, as shown in Figure S1b and Equation (b) below:

Synthesis of ZnO :

$Zn^{2+} + 2OH^- \rightarrow Zn(OH)_2 \rightarrow ZnO + H_2O$ (at 90 °C)

Selective etching of ZnO :

$ZnO + H_2O \rightarrow Zn(OH)_2 \rightarrow Zn^{2+} + 2OH^-$ (at 40 °C)

Meanwhile, the six sidewalls of the ZNR are nonpolar planes, which are known as the most chemically stable planes in the ZNR structure. Therefore, the etching rate of the top surface of the ZNR, which is the metastable Zn polar plane, is much faster than that of the ZNR sidewalls, resulting in the focal dissolution of the ZNR core [1].

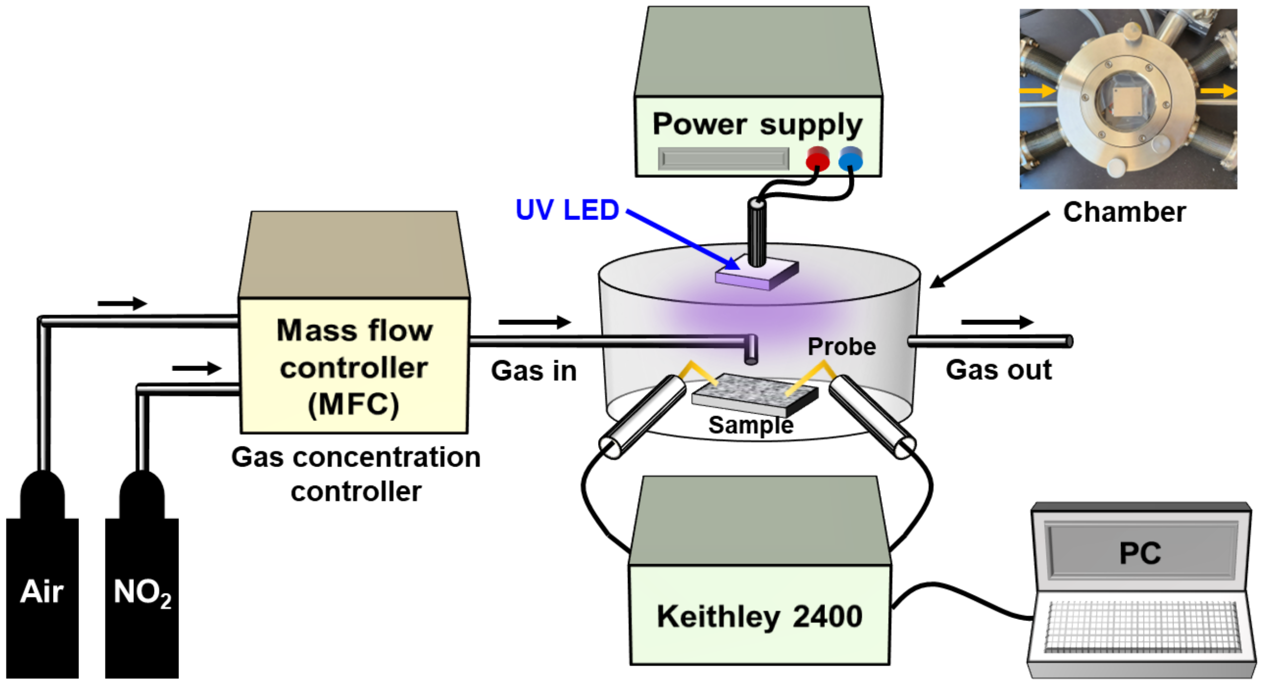

Figure S2. The gas-sensing measurement system is illustrated in a schematic image. The NO_2 gas concentration was controlled by two mass flow controllers in the gas concentration controller for air and NO_2 gas. The air and NO_2 gas were then injected into a homemade gas chamber. In all measurements, NO_2 gas for measurement was injected after stabilizing the chamber atmosphere with forming air for 30 minutes. The samples were connected to a Keithley 2400 source meter via probes in the chamber. A UV LED connected by a power supply in the chamber irradiated UV light onto the samples. The resistance signal from the samples was read by the source meter and transmitted in real time through the LabVIEW program on the PC.

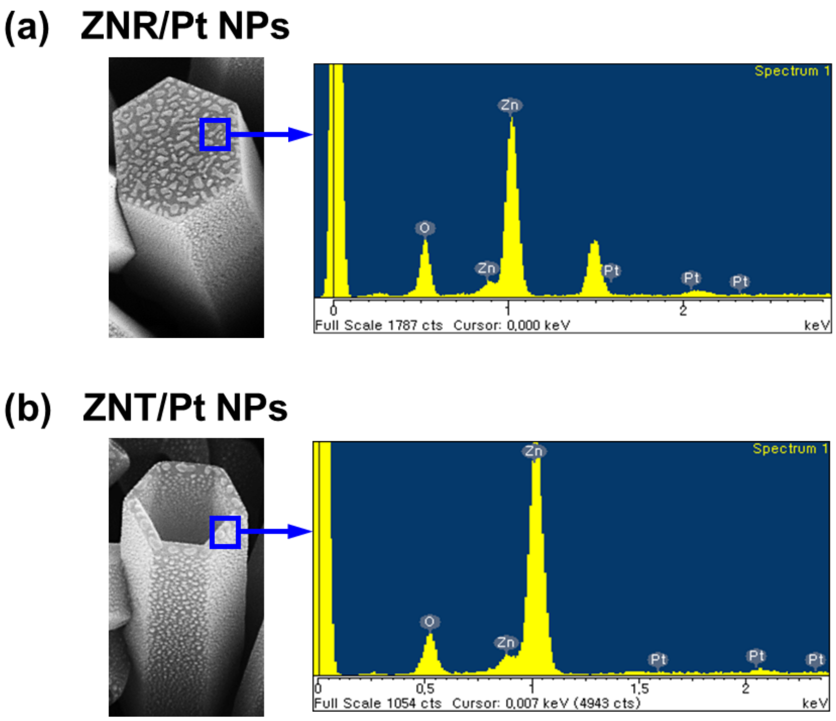

Figure S3. SEM-EDS results of ZNR/Pt NPs and ZNT/Pt NPs. It was confirmed that both the ZNR/Pt NPs and ZNT/Pt NPs samples were composed of Zn, O, and Pt.

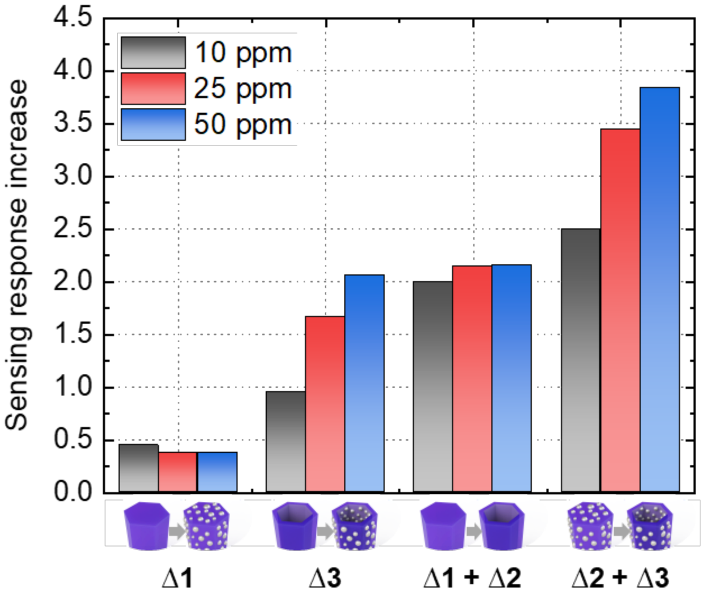

Figure S4. Plot of change in gas-sensing responsivity according to structural changes. In this plot, the y-axis shows how much the sensing response has improved. In case that Pt NPs were formed on ZNR and ZNT structures, the sensing responses increased more than in case that Pt NPs were not formed on ZNR and ZNT structures. Moreover, the increase of sensing response due to Pt NPs was greater with ZNT than with ZNR. In addition, in case that the structural change from ZNR to ZNT occurred, the increase in sensing responsivity tended to be greater than in case that Pt NPs were formed.

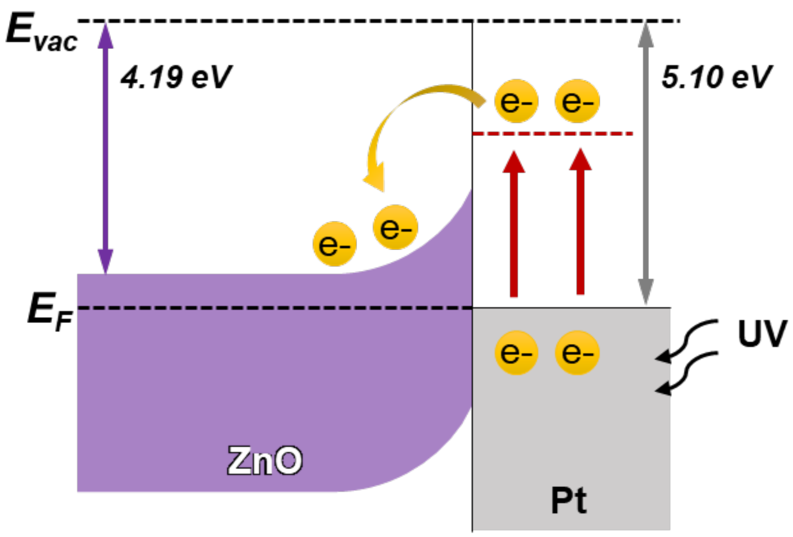

Figure S5. Energy bandgap diagram of ZnO and Pt in Schottky junction. Hot electrons are generated under UV light and injected into ZnO. The hot electrons could not transfer back to the Pt NPs from ZnO because of the Schottky barrier formed at the interface between the Pt NPs and ZnO.

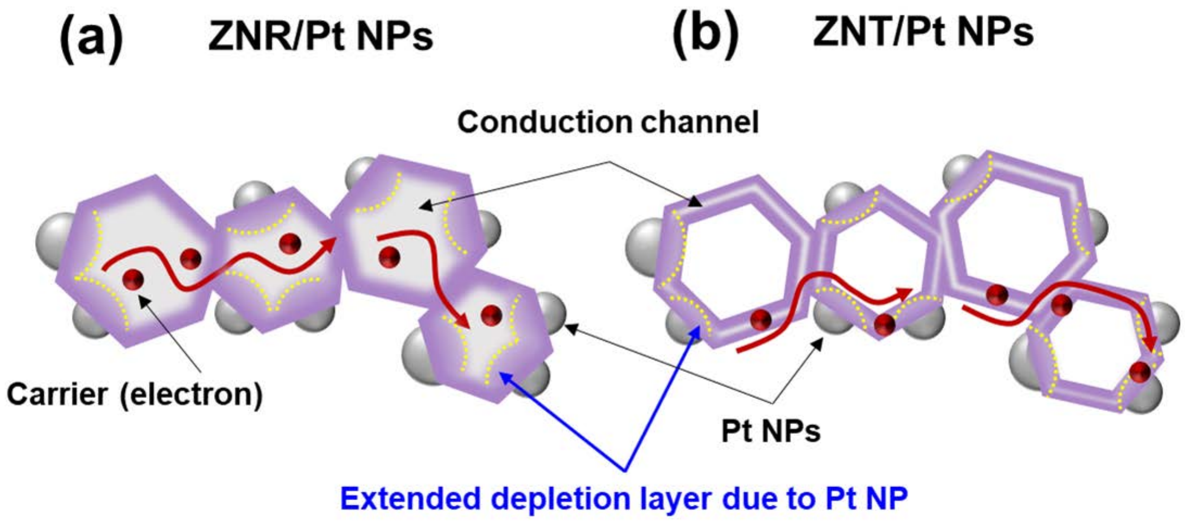

Figure S6. Schematic images of increased depletion layer width of ZNRs and ZNTs due to Pt NPs formation.

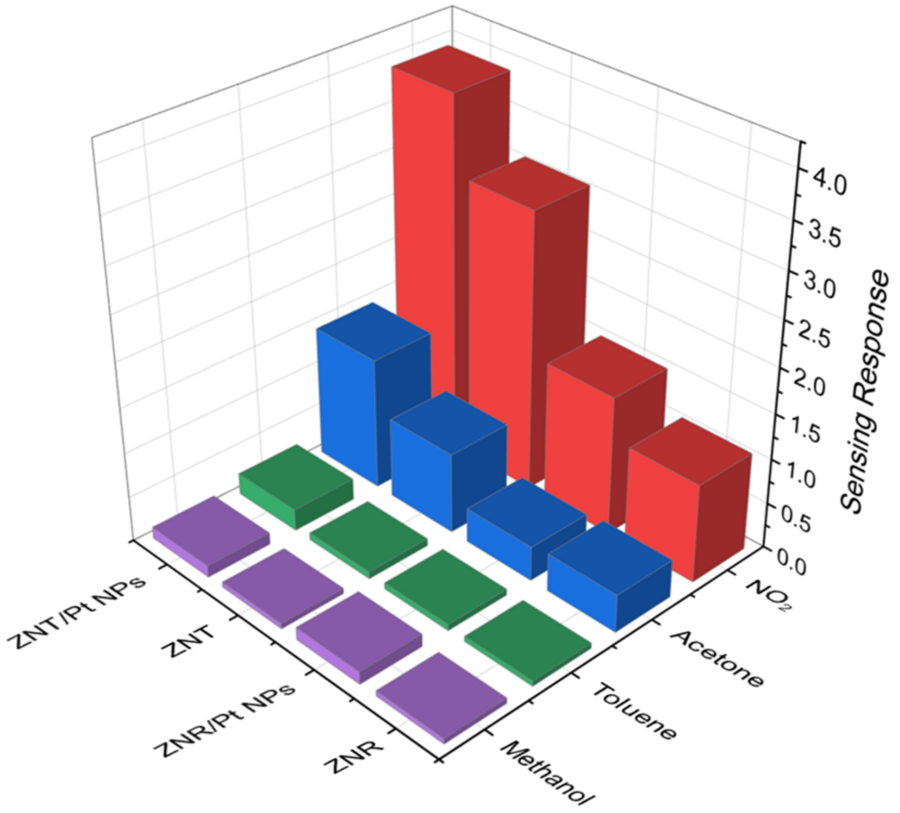

	NO_2 (10ppm)	Toluene (50ppm)	Acetone (250ppm)	Methanol (100ppm)
**ZNR**	1.09	0.06	0.42	0.06
**ZNR/Pt NPs**	1.54	0.07	0.38	0.14
**ZNT**	3.09	0.08	0.89	0.06
**ZNT/Pt NPs**	3.92	0.24	1.46	0.13

Figure S7. Sensing responsivity graph for NO_2, acetone, toluene, and methanol gases in different gas concentrations. All samples were confirmed to have the highest response in NO_2 gas.

*References

[1] Gan, X.; Li, X.; Gao, X.; Yu, W. Investigation on chemical etching process of ZnO nanorods toward nanotubes. *J. Alloys Compd.* **2009**, *481*, 397–401.
